# Translating Clinical Questions by Physicians Into Searchable Queries: Analytical Survey Study

**DOI:** 10.2196/16777

**Published:** 2020-04-20

**Authors:** Aurélie Seguin, Robert Brian Haynes, Sebastian Carballo, Alfonso Iorio, Arnaud Perrier, Thomas Agoritsas

**Affiliations:** 1 Division of General Internal Medicine Department Medicine University Hospitals of Geneva Geneva Switzerland; 2 Department of Health Research Methods, Evidence, and Impact McMaster University Hamilton, ON Canada

**Keywords:** evidence-based medicine, evidence retrieval, Web-based resources, search engines, search taxonomy, clinical information science

## Abstract

**Background:**

Staying up to date and answering clinical questions with current best evidence from health research is challenging. Evidence-based clinical texts, databases, and tools can help, but clinicians first need to translate their clinical questions into searchable queries. MacPLUS FS (McMaster Premium LiteratUre Service Federated Search) is an online search engine that allows clinicians to explore multiple resources simultaneously and retrieves one single output that includes the following: (1) evidence from summaries (eg, UpToDate and DynaMed), (2) preappraised research (eg, EvidenceAlerts), and (3) non-preappraised research (eg, PubMed), with and without validated bibliographic search filters. MacPLUS FS can also be used as a laboratory to explore clinical questions and evidence retrieval.

**Objective:**

Our primary objective was to examine how clinicians formulate their queries on a federated search engine, according to the population, intervention, comparison, and outcome (PICO) framework. Our secondary objective was to assess which resources were accessed by clinicians to answer their questions.

**Methods:**

We performed an analytical survey among 908 clinicians who used MacPLUS FS in the context of a randomized controlled trial on search retrieval. Recording account log-ins and usage, we extracted all 1085 queries performed during a 6-month period and classified each search term according to the PICO framework. We further categorized queries into background (eg, “What is porphyria?”) and foreground questions (eg, “Does treatment A work better than B?”). We then analyzed the type of resources that clinicians accessed.

**Results:**

There were 695 structured queries, after exclusion of meaningless queries and iterations of similar searches. We classified 56.5% (393/695) of these queries as background questions and 43.5% (302/695) as foreground questions, the majority of which were related to questions about therapy (213/695, 30.6%), followed by diagnosis (48/695, 6.9%), etiology (24/695, 3.5%), and prognosis (17/695, 2.5%). This distribution did not significantly differ between postgraduate residents and medical faculty physicians (*P*=.51). Queries included a median of 3 search terms (IQR 2-4), most often related to the population and intervention or test, rarely related to the outcome, and never related to the comparator. About half of the resources accessed (314/610, 51.5%) were summaries, 24.4% (149/610) were preappraised research, and 24.1% were (147/610) non-preappraised research.

**Conclusions:**

Our results, from a large sample of real-life queries, could guide the development of educational interventions to improve clinicians’ retrieval skills, as well as inform the design of more useful evidence-based resources for clinical practice.

**Trial Registration:**

ClinicalTrials.gov NCT02038439; https://www.clinicaltrials.gov/ct2/show/NCT02038439

## Introduction

Web-based searches have become the norm when looking for information and answers to most of our questions in daily life. This has also become true in the practice of medicine; online medical resources to access evidence are increasingly considered “as essential as the stethoscope” [1]. While famous search engines, such as Google, or information sources, such as Wikipedia, are used in both medical and nonmedical worlds, answering clinical questions to inform point-of-care decisions has additional challenges and implications [2]. Triggered by more than 20 years of evidence-based medicine (EBM) [3,4], the unit of information in medicine comes mostly in the form of research evidence, published across thousands of medical journals and indexed in numerous databases (eg, MEDLINE, Embase, and the Cumulative Index to Nursing and Allied Health Literature [CINAHL]). The volume of this new evidence through all these channels is rapidly increasing at the pace of 3000-4000 new publications per day, compiled or processed in hundreds of EBM summaries and resources [5-7].

**Figure 1:**
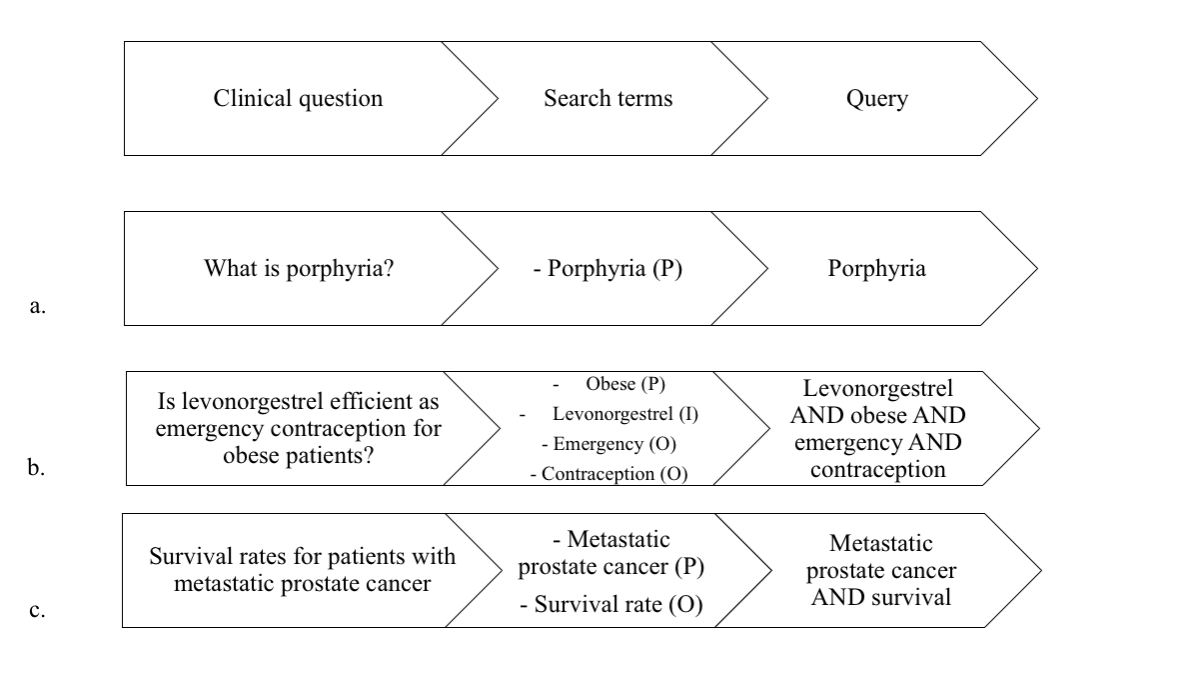
The path from a clinical question to a query using the population (P), intervention (I), comparison (C), and outcome (O) (PICO) framework. Examples are shown for (a) a background question, (b) a foreground therapy question, and (c) a foreground prognosis question.

Physicians are typically familiar with only a few of these resources, likely those to which they have been exposed in training or by peers, and often ignore most of the ecosystem and architecture of published evidence. Yet, their daily practice triggers, on average, five to eight questions every 10 patients [8-10]. Clinical questions can be classified as background and foreground questions (see Figure 1). Background questions (eg, “What is porphyria?”) are typically about the nature of a disorder, a measure, a treatment, or a test. They are easily answered through online textbooks. Foreground questions are more directly related to the diagnosis, prognosis, and treatment of a given patient population (eg, “How effective would levonorgestrel be as emergency contraception for an obese patient?”) [11]. The teaching of EBM recommends that foreground questions be formulated according to the population, intervention, comparison, and outcome (PICO) framework, or the population, exposure, comparison, and outcome (PECO) framework, and answered by research evidence [12].

How physicians translate their clinical questions into searchable queries remains poorly known. How many search terms do they use? How often do their queries fit the PICO framework [12,13]? Do experienced and fully trained clinicians differ from residents in training? Do queries differ according to the medical specialty? We aimed to examine these questions in a large sample of practicing clinicians of various levels of training and specialty type.

The type of search engine or evidence resource may also influence the way we conduct queries. Google and Wikipedia tend to retrieve relevant answers, albeit selective, with intuitive, less-structured search strategies [14-16]. Some EBM online textbooks and evidence summaries may provide a similar user experience to clinicians. By contrast, searching PubMed or other databases requires more training and structure, is less intuitive, and tends to produce large and diluted outputs for similar clinical questions [12].

**Figure 2:**
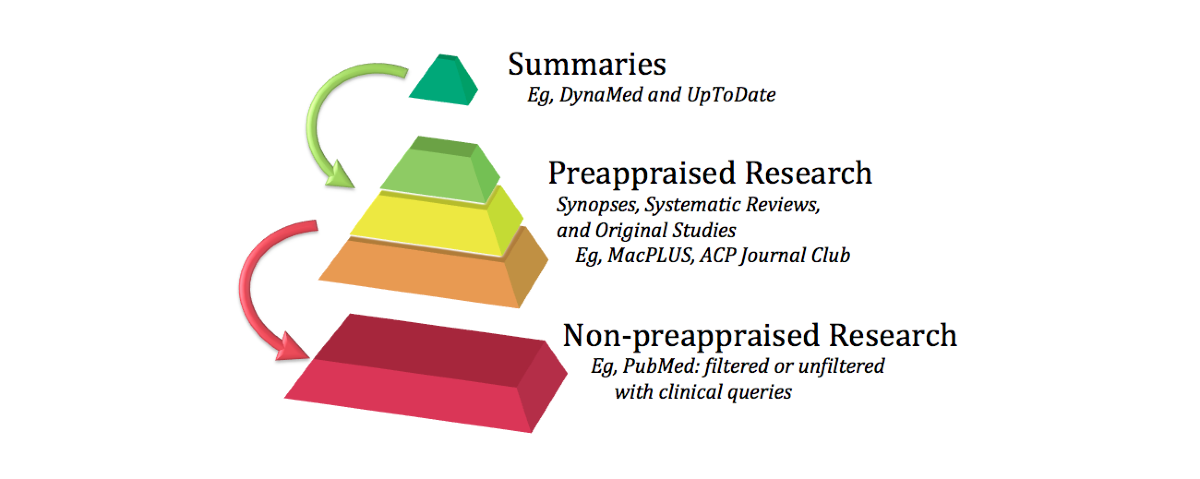
Synopses, systematic reviews, and select studies of evidence-based medicine resources provided in the federated search engine MacPLUS FS (McMaster Premium LiteratUre Service Federated Search); adapted from Agoritsas et al, 2014. ACP: American College of Physicians.

We, therefore, explored how clinicians formulate their queries in a federated online search engine, namely MacPLUS FS (McMaster Premium LiteratUre Service Federated Search). MacPLUS FS allows clinicians to explore multiple resources simultaneously, retrieving one single output that includes the following: (1) evidence from evidence-based summaries (eg, UpToDate and DynaMed), (2) preappraised research (eg, EvidenceAlerts), and (3) non-preappraised research (eg, PubMed), with and without validated search filters (see Figure 2). In this study, we will outline how we used MacPLUS FS, which functions as a laboratory, to explore clinical questions, the taxonomy of queries, and evidence retrieval (ie, what resources clinicians access to answer their questions when provided with a wide array of EBM resources) (see Multimedia Appendix 1) [5]. While MacPLUS FS functions as a laboratory for evidence retrieval research, its exact twin—ACCESSSS search engine—is freely available online [17].

## Methods

### Study Design and Clinician Sample

We conducted an analytical survey of clinical search queries among 431 postgraduate medical trainees and 477 medical faculty members registered to a federated search engine, MacPLUS FS. The service was freely available to registered users from any computer with an internet browser throughout the clinical setting or elsewhere.

Participating clinicians consented to be enrolled in 6-month, MacPLUS FS, randomized controlled trials [5], which tested three interventions to enhance the quantity and quality of searching for current best evidence in order to answer clinical questions in a factorial design. As described with more detail in the published protocol of the trials [5], we tested the following three interventions embedded in MacPLUS FS: (1) a Web-based clinical question recorder, (2) an evidence retrieval coach composed of eight short educational videos, and (3) an audit, feedback, and gamification approach to evidence retrieval, based on the allocation of *badges* and *reputation scores*. Participating clinicians were randomized to each of the three interventions in a factorial design (A × B × C).

For each clinician, utilization of MacPLUS FS was recorded through accounts tracking log-ins and usage, including their detailed search queries. Registration to the service was free, and access to each evidence resource was through clinicians’ academic institutions, mostly McMaster University, Hamilton, Canada. Clinicians were categorized according to their baseline search levels and specialty types [5].

### Sample of Search Queries

We extracted all 1085 search queries performed by clinicians during the conduct of the MacPLUS FS trials. Two authors (AS and TA) assessed each query individually, counting the number of search terms—counting all words (eg, the query “porphyria” contains 1 term)—and documenting all abbreviations and Boolean terms (ie, logical operators such as “AND,” “OR,” or “NOT”). Search queries were then classified into (1) structured searches, (2) searches for specific articles (eg, when clinicians typed in the title of a given study), (3) iteration of structured searches, namely a group of related structured queries with a similar PICO question within the same log-in session, and (4) undetermined searches (eg, “Scimitar”).

### Assessment of Search Queries and Evidence Resources Access

The same two authors (AS and TA) classified structured queries into background or foreground questions (see Figure 1), according to the PICO framework, and blinded the participants’ characteristics, except the log-in session. Queries that included only terms related to population or intervention were classified as background questions. Those including several terms related to population and intervention and/or outcome and/or comparator were categorized as foreground, and further categorized into therapy, diagnosis, etiology, and prognosis. For each query, we examined the distribution of access to each evidence resource from the federated search: *summaries*, *preappraised research*, and *non-preappraised research* (see Figure 2).

### Statistical Analysis

We examined types of questions (ie, background, foreground, and type of foreground) according to the level of training as well as clinicians’ specialties and baseline frequencies of search (ie, in the prior months since their registration to MacPLUS FS). We then examined the number and type of search terms across each type of question. We compared distributions using chi-square parametric tests when relevant and Kruskall-Wallis tests for nonnormal distributions. Data abstraction was done using Microsoft Excel 2016, version 15.29, and data analysis was performed using SPSS Statistics for Windows, version 23.0 (IBM Corp).

## Results

### Clinicians

Participants were postgraduate residents and medical faculty members who had registered in MacPLUS FS prior to the trial. Of the 678 postgraduate residents and 753 medical faculty members, 431 (63.6%) and 477 (63.3%), respectively, were deemed eligible after the exclusion of 247 postgraduate residents and 266 medical faculty members, who either never logged in to MacPLUS FS during the year prior to the study or quit the institutions served by MacPLUS FS [5]. Searchers were further classified, depending on their baseline average search frequencies during the 6 months prior to the trial [5], as *regular searchers* (≥1 search per month), *occasional searchers* (<1 search per month), or *alert-only users* (no searches).

### From Clinicians to Queries

The 908 clinicians made 1085 search queries, of which 235 (21.66%) were subsequent iterations of the same search, 124 (11.43%) were a search for a specific article, and 31 (2.86%) could not be classified and remained undetermined. A total of 695 out of 1085 queries (64.06%) were structured queries following the PICO format, with 480 out of 695 (69.1%) single queries, whereas 215 (30.9%) included a group of related queries. This corresponds to an average of 2.1 attempts per group query.

Table 1 summarizes the distributions of the 695 structured queries. We classified 56.5% (393/695) as background and 43.5% (302/695) as foreground questions, the majority of which were related to therapy (213/695, 30.6%), followed by diagnosis (48/695, 6.9%), etiology (24/695, 3.5%), and prognosis (17/695, 2.4%). Distributions did not differ according to level of training (*P*=.51) (see Table 1).

**Table 1:** Type of structured queries according to level of training.

Query type		Level of training, n (%)		
		Postgraduate residents (n=409)	Medical faculty members (n=286)	Total (n=695)^a^
Background		239 (58.4)	154 (53.8)	393 (56.5)
**Foreground**		170 (41.6)	132 (46.2)	302 (43.5)
	Therapy	112 (27.4)	101 (35.3)	213 (30.6)
	Diagnosis	34 (8.3)	14 (4.9)	48 (6.9)
	Etiology	15 (3.7)	9 (3.1)	24 (3.5)
	Prognosis	9 (2.2)	8 (2.8)	17 (2.4)
Total		409/695 (58.8)	286/695 (41.2)	695 (100)

^a^There were 695 structured queries among 1085 queries, the remaining being 235 iterations of the same search, 124 specific article searches, and 31 undetermined searches.

Table 2 shows the distributions of queries related to background and foreground clinical questions, with respect to the clinicians’ levels of training, specialty types (ie, family medicine, internal medicine, internal medicine specialties, pediatrics, psychiatry, surgery, anesthesiology, and others detailed in Multimedia Appendix 2), and categories of search frequency. Internal and family medicine physicians made 48.5% (337/695) of structured queries, 55.2% (186/337) of which were related to background content (see Table 2). However, there were differences regarding the frequencies of searches with regular searchers looking for significantly more background questions (*P*=.009). There were no differences between specialty types (*P*=.67).

**Table 2:** Background versus foreground queries with respect to characteristics of clinicians.

Characteristic		Question type, n (%)		
		Background	Foreground	Total
**Training**				
	Postgraduate residents	239 (58.4)	170 (41.6)	409 (100)
	Medical faculty members	154 (53.8)	132 (46.2)	286 (100)
**Specialty type **				
	Family medicine	114 (54.0)	97 (46.0)	211 (100)
	Internal medicine	72 (57.1)	54 (42.9)	126 (100)
	Other specialties^a^	207 (57.8)	151 (42.2)	358 (100)
**Categories of search frequency**				
	≥1 (regular searchers)	164 (62.8)	97 (37.2)	261 (100)
	<1 (occasional searchers)	88 (51.8)	82 (48.2)	170 (100)
	0 (alert-only users)	141 (53.4)	123 (46.6)	264 (100)
Total		393 (56.5)	302 (43.5)	695 (100)

^a^Other specialties includes internal medicine specialties, pediatrics, psychiatry, surgery, anesthesiology, and others detailed in Multimedia Appendix 2.

Table 3 details the components of the queries. Queries included a median number of 3 search terms (IQR 2-4). There were significantly more terms with foreground questions compared to background questions (*P*<.001). Indeed, there were 70.2% (276/393) of background questions with 2 or fewer terms versus 18.2% (55/302) of the foreground questions, and 81.8% (247/302) of foreground questions with 3 or more terms versus 29.8% (117/393) of the background questions.

Overall, 72.5% (504/695) of structured queries (see Table 3) contained at least 1 term related to population, and 45.9% (319/695) contained at least 1 term related to an intervention. Few queries contained terms about etiology, diagnostic tests, or outcome. No query included the comparator. Background queries included a median of 2 search terms (IQR 1-3). Of these queries, 71.2% (280/393) included a population term, 24.7% (97/393) included an intervention term, 1.0% (4/393) included an etiology term, 6.1% (24/393) included a diagnostic term, and 2.5% (10/393) included an outcome term. Foreground queries included a median of 4 search terms (IQR 3-5). Of these queries, 74.2% (224/302) included a population term, 73.5% (222/302) included an intervention term, 21.5% (65/302) included an outcome term, 16.2% (49/302) included a diagnostic term, and 7.6% (23/302) included an etiology term. Clinicians made no use of explicit Boolean search terms to link various PICO elements.

**Table 3:** Number of terms with respect to type of structured queries.

Type of query		Number of terms, median (IQR)	Distribution of terms^a^ within each type of query, n (%)				
			≥1 population term	≥1 intervention term	≥1 etiology term	≥1 diagnostic term	≥1 outcome term
Background (n=393)		2 (1-3)	280 (71.2)	97 (24.7)	4 (1.0)	24 (6.1)	10 (2.5)
**Foreground**							
	All foreground (n=302)	4 (3-5)	224 (74.2)	222 (73.5)	23 (7.6)	49 (16.2)	65 (21.5)
	Intervention (n=213)	3 (3-5)	173 (81.2)	210 (98.6)	1 (0.5)	2 (0.9)	43 (20.2)
	Diagnostic (n=48)	4 (2-5)	24 (50)	6 (13)	0 (0)	46 (96)	0 (0)
	Etiology (n=24)	4 (3-5)	12 (50)	2 (8)	22 (92)	0 (0)	17 (71)
	Prognostic (n=17)	5 (4-5)	15 (88)	4 (24)	0 (0)	1 (6)	5 (29)
Total structured queries (n=695)		3 (2-4)	504 (72.5)	319 (45.9)	27 (3.9)	73 (10.5)	75 (10.8)

^a^The distribution of terms is significantly different between background and foreground queries (*P*<.001). No query included the comparator search term in either type of query, so we did not include a column for the comparator term.

The number of evidence-based resources that clinicians accessed for each type of query (ie, by clicking the available links in the search output) are displayed in Table 4. The distribution of accessed resources is significantly different across categories (*P*<.001). Although 35.7% (248/695) of structured queries did not result in any resource access, 39.9% (277/695) led to one resource accessed, 11.8% (82/695) led to two, and 12.7% (88/695) led to three or more resources. Across all 1085 queries, the average number of resources accessed was 0.88 (SD 1.42). When users attempted a second search on the same clinical question (ie, similar PICO concepts but revised search terms), 7.2% (17/235) resulted in one or more resources accessed, while 92.8% (218/235) led to an end of their search query with no additional resource accessed. When searching for a specific article, 37.9% (47/124) led to one resource accessed and 12.9% (16/124) led to two or more resources accessed.

**Table 4:** Number of accessed sites across 1085 queries.

Accessed site	Query type, n (%)				
	Structured search (n=695)	Iteration of a structured search^a^ (n=235)	Specific article search (n=124)	Undetermined search (n=31)	Total (N=1085)
0	248 (35.7)	218 (92.8)	61 (49.2)	22 (71)	549 (50.60)
1	277 (39.9)	12 (5.1)	47 (37.9)	5 (16)	341 (31.43)
2	82 (11.8)	3 (1.3)	13 (10.5)	1 (3)	99 (9.12)
3	45 (6.5)	1 (0.4)	1 (0.8)	2 (6)	49 (4.52)
4	15 (2.2)	1 (0.4)	1 (0.8)	1 (3)	18 (1.66)
≥5	28 (4.0)	0 (0)	1 (0.8)	0 (0)	29 (2.67)
Total	695 (100)	235 (100)	124 (100)	31 (100)	1085 (100)

^a^A group of related structured queries with similar population, intervention, comparison, and outcome (PICO) concepts, but revised search terms within the same log-in session.

Table 5 shows types of accessed resources with respect to level of training, type of query, and specialty. Across the 695 structured queries, there were 610 accessed resources, with half of them (314/610, 51.5%) being summaries, 24.4% (149/610) being preappraised research, and 24.1% (147/610) being non-preappraised research. When comparing the distribution of resources that were accessed across the federated search output, medical faculty members looked at significantly more summaries than did postgraduate trainees (*P*<.001), and family physicians looked at significantly more resources than did internists and specialized physicians (*P*<.001).

**Table 5:** Resources accessed across structured queries that led to at least one evidence resource.

Training and specialty		Resources accessed^a^, n (%)				*P* value
		Summaries	Preappraised research	Non-preappraised research	Total	
**Training**						**<.001**
	Postgraduate residents	150 (43.5)	85 (24.6)	110 (31.9)	345 (100)	
	Medical faculty members	164 (61.9)	64 (24.2)	37 (14.0)	265 (100)	
**Specialty**						**.001**
	Family medicine	103 (64.0)	31 (19.3)	27 (16.8)	161 (100)	
	Internal medicine	60 (46.2)	41 (31.5)	29 (22.3)	130 (100)	
	Other specialty	151 (47.3)	77 (24.1)	91 (28.5)	319 (100)	
Total		314 (51.5)	149 (24.4)	147 (24.1)	610 (100)	

^a^A total of 610 resources were accessed by 695 structured queries. Access was recorded through the number of links accessed from MacPLUS (McMaster Premium LiteratUre Service) search output.

## Discussion

### Principal Findings

Among 1085 queries made by 908 clinicians, 695 were structured queries. A small majority were related to background questions, and most foreground questions were questions about therapy, rather than diagnostic or prognostic questions. Structured queries included a median of 3 terms, most often related to the population and intervention or test, rarely related to the outcome, and never related to the comparator. Explicit Boolean terms were rarely used; of note, the search engine assumed by default a Boolean “AND” between search terms. About half of the resources accessed were summaries, while the rest were equally divided between preappraised and non-preappraised resources.

We found no difference between searches made by postgraduate resident trainees and medical faculty members. As they are in training, one could have expected postgraduate residents to have more background questions, whereas faculty members were expected to have more foreground questions, for example, in comparing the effectiveness or risks of management strategies. Our results did not confirm this assumption, as faculty members had more than half of their searches on background questions as well. This may be due to the complexity of patient care. A given faculty member may be an expert in a given field but adopt a learning strategy to rapidly get the big picture, to understand uncommon situations. Their high level of access for summary resources, such as UpToDate or DynaMed, likely supports this explanation. Similarly, family doctors also accessed more summary resources, not only because of their need for quick clear answers to questions arising within short appointments with patients, but also, perhaps, because they provide care for patients across an entire age spectrum.

Another issue relates to the frequency of searches clinicians are able to perform in daily life. In our study, 908 clinicians performed only 1085 queries in 6 months. Other studies have shown that clinicians tend not to search in order to answer the questions that arise in their daily clinical practice [10,18-20]. In our study, a third of the structured searches led to no resource access through the platform, for which we have no explanation. More than 20 years ago, Ely et al [19,20] already showed that clinicians spend less than 2 minutes looking to answer a question—a finding probably even more accurate nowadays with increased access to information online—and suggested that searching for evidence may not fit with clinicians’ multiple tasks and training [21]. It is also possible that clinicians have looked for answers in other resources (eg, PubMed or UpToDate), or even in Google, Google Scholar, or Wikipedia. Alternatively, clinicians may often not conduct searches online but, rather, directly ask their peers or use local guidelines [22-26]. Reasons include convenience and time constraints to access ready-to-care information that conforms with local knowledge rather than challenging it. Although looking for answers on a general search engine or via colleagues or guidelines is easier, it does not guarantee or promote a fully EBM approach to health care [27,28]. Clinicians could, therefore, benefit from information specialists available to help at the point of care [29] and from the design of more intuitive tools to navigate the complexity of the evidence ecosystem.

Another observation from our study is that clinicians’ queries tend to remain relatively simple: few search terms, often covering few PICO concepts, mostly population and intervention. While simple strategies work well for high-level summaries, they are much less efficient with large databases like PubMed. Our daily habits for searching on the Web may explain clinicians’ tendencies for simple queries. Strictly from a user’s perspective, we have all become very efficient in searching for information mostly through Google and Wikipedia, just by typing a few intuitive keywords in the free-text bar at the top of a webpage. Medical search engines may misguide the user in having them assume the engine will work similarly to Google [30].

One area for improvement of search engines could be to invite users to structure their queries according to the PICO framework. Schardt et al [31] have found that searchers using the PICO format had more precise results than users searching with the standard interface on PubMed; in that study, precision scores were defined as the number of relevant or gold-standard articles retrieved in a result set compared to the total number of articles retrieved in that set. Unfortunately, and possibly due to the small sample, the difference between the search groups was not statistically significant [31]. An alternative could be to improve search engine functionalities, with the remaining challenge, however, of avoiding any cherry picking of the evidence and, thus, potentially biased conclusions for clinical practice. A potential solution lies with federated search engines like MacPLUS FS, which complement summary-level evidence with other preappraised and non-preappraised research. Indeed, we have shown that physicians access all types of resources translating an interest into different layers of the EBM when these layers are displayed together on one page (see Figure 2). The use of a federated search engine may thus help clinicians navigate across EBM resources, allowing them to look at and compare different resources simultaneously and to identify the current best evidence that is most adapted to their information needs.

### Limitations and Strengths of the Study

The main limitation of our study was that clinicians likely used other means than MacPLUS FS to answer some of their daily questions. Our design also did not assess the clinical impact of the answers retrieved. This would have required mixed methods approaches to estimate the number of patients needed to benefit from information (ie, number needed to benefit from information [NNBI]), as described by Pluye et al [32].

Finally, our sample of searches was recorded in the context MacPLUS FS randomized controlled trials [5], and it remains unclear how search queries may have differed without the possible influence of the interventions tested. The second intervention— the evidence retrieval coach—included eight short educational videos, of which only one was providing advice on the PICO formulation of clinical queries. However, only a small group of participants would have been exposed to that short video, and none of the other interventions were specifically aimed at improving the formulation of queries.

Strengths include the direct record of queries in one of the largest samples of physicians from different specialties and levels of practice. It is also the first study on a federated search engine, which allowed us to show that clinicians access all resources and not only summary-level evidence.

### Conclusions

A constant flow of new articles overwhelms clinicians who are continuously exposed to them. To keep up and to answer our clinical questions, it is essential to clarify and translate clinical questions into searchable queries. Our results could lead to the development of educational and clinical interventions on how to increase searching skills [2]. These could include workshops and tools to translate clinical questions into queries and to better structure and adapt them to each type of resource.

Our findings also highlight the potential role of federated search engines over the use of single resources to meet clinicians’ needs [23]. A federated search engine retrieves evidence and may help clinicians get answers to their questions with current best evidence, even with short time frames and limited experience and skills for searching.

Other avenues of research include the improvement of search functionalities and clinical interventions to meet users’ expectations in navigating through the evidence, in order to rapidly find the most relevant and least-biased answers for better clinical practice and patient care.

## Appendix

Multimedia Appendix 1 Evidence-based medicine (EBM) resources accessible through MacPLUS FS (McMaster Premium LiteratUre Service Federated Search).

Multimedia Appendix 2 Characteristics of clinicians by specialty types.

## Abbreviations

ACP: American College of Physicians
CINAHL: Cumulative Index to Nursing and Allied Health Literature
EBM: evidence-based medicine
MacPLUS FS: McMaster Premium LiteratUre Service Federated Search
NNBI: number needed to benefit from information
PECO: population, exposure, comparison, and outcome
PICO: population, intervention, comparison, outcome
