# Supplementary material for: Translating Clinical Questions by Physicians Into Searchable Queries: Analytical Survey Study
Source: JMIR Med Educ. 2020 Apr 20;6(1):e16777. doi: 10.2196/16777 (PMC7199131; doi:10.2196/16777)
Supplement: Multimedia Appendix 1 [file mededu_v6i1e16777_app1.doc]

**Multimedia Appendix 1.** EBM Resources accessible through MacPLUS Federated Search.

|  |  | |  |
| --- | --- | --- | --- |
| **Summaries** | | DynaMed UpToDate Best Practice ACP PIER | |
|
|
|
| **Preappraised research** | |  | |
| Synopses of systematic reviews | | ACP Journal Club,  Database of Abstracts of Reviews of Effects (DARE) | |
|
| Systematic reviews | | McMasterPLUS (including Cochrane) | |
| Synopses of studies | | McMasterPLUS | |
| **Non-preappraised research** | |  | |
| Filtered studies | | Clinical Queries in PubMed | |
| Unfiltered studies | | PubMed (MEDLINE) | |

^a^ At the time of the data collection. Adapted from Table 1 in Agoritsas et al. [5].
