# Supplementary material for: Translating Clinical Questions by Physicians Into Searchable Queries: Analytical Survey Study
Source: JMIR Med Educ. 2020 Apr 20;6(1):e16777. doi: 10.2196/16777 (PMC7199131; doi:10.2196/16777)
Supplement: Multimedia Appendix 2 [file mededu_v6i1e16777_app2.doc]

**Multimedia Appendix 2.** Characteristics of clinicians by specialty types.

| **Specialty type** - n (%) |  |
| --- | --- |
| Anaesthesiology | 29 (3.2) |
| Anatomical Pathology | 5 (0.6) |
| Basic Clinical Training | 2 (0.2) |
| Cardiology | 34 (3.7) |
| Clinical Immunology (Adult & Paediatric) | 11 (1.2) |
| Clinician Investigator Program | 1 (0.1) |
| Community Medicine | 7 (0.8) |
| Critical Care | 20 (2.2) |
| Dermatology | 2 (0.2) |
| Developmental Pediatrics | 3 (0.3) |
| Diagnostic Radiology | 6 (0.7) |
| Emergency Medicine, Royal College Stream | 24 (2.6) |
| Emergency Medicine/Family Medicine Stream | 30 (3.3) |
| Family Medicine | 237 (26.1) |
| Gastroenterology | 19 (2.1) |
| General Pathology | 1 (0.1) |
| General Surgery | 24 (2.6) |
| Geriatric Medicine | 17 (1.9) |
| Gynaecological Oncology | 2 (0.2) |
| Haematology (Adult) | 17 (1.9) |
| Haematology-Oncology (Pediatric) | 1 (0.1) |
| Infectious Diseases | 9 (1) |
| Internal Medicine | 131 (14.4) |
| Medical Biochemistry | 3 (0.3) |
| Medical Microbiology | 1 (0.1) |
| Medical Oncology | 8 (0.9) |
| Neonatal-Perinatal Medicine | 12 (1.3) |
| Nephrology | 4 (0.4) |
| Neurology | 15 (1.7) |
| Neurosurgery | 10 (1.1) |
| Obstetrics and Gynecology | 11 (1.2) |
| Ophthalmology | 4 (0.4) |
| Orthopedic Surgery | 13 (1.4) |
| Otolaryngology- Head and Neck | 5 (0.6) |
| Pediatric Gastroenterology | 1 (0.1) |
| Pediatric Neurology | 1 (0.1) |
| Pediatrics | 55 (6.1) |
| Physical Medicine & Rehabilitation | 15 (1.7) |
| Plastic Surgery | 6 (0.7) |
| Psychiatry | 45 (5) |
| Radiation Oncology | 4 (0.4) |
| Respirology | 17 (1.9) |
| Rheumatology | 12 (1.3) |
| Thoracic Surgery | 1 (0.1) |
| Transfusion Medicine | 1 (0.1) |
| Trauma | 2 (0.2) |
| Urology | 4 (0.4) |
| Vascular Surgery | 4 (0.4) |
| No Data | 22 (2.4) |
| **Total number of clinicians** | **908 (100)** |
